# Dynamic studies of H-Ras•GTPγS interactions with nucleotide exchange factor Sos reveal a transient ternary complex formation in solution

**DOI:** 10.1038/srep29706

**Published:** 2016-07-14

**Authors:** Uybach Vo, Navratna Vajpai, Kevin J. Embrey, Alexander P. Golovanov

**Affiliations:** 1 Manchester Institute of Biotechnology and Faculty of Life Sciences, The University of Manchester, 131 Princess Street, Manchester M1 7DN, UK; 2 AstraZeneca, Discovery Sciences, Mereside, Alderley Park, Cheshire, SK10 4TF, UK

## Abstract

The cycling between GDP- and GTP- bound forms of the Ras protein is partly regulated by the binding of Sos. The structural/dynamic behavior of the complex formed between activated Sos and Ras at the point of the functional cycle where the nucleotide exchange is completed has not been described to date. Here we show that solution NMR spectra of H-Ras∙GTPγS mixed with a functional fragment of Sos (Sos^Cat^) at a 2:1 ratio are consistent with the formation of a rather dynamic assembly. H-Ras∙GTPγS binding was in fast exchange on the NMR timescale and retained a significant degree of molecular tumbling independent of Sos^Cat^, while Sos^Cat^ also tumbled largely independently of H-Ras. Estimates of apparent molecular weight from both NMR data and SEC-MALS revealed that, at most, only one H-Ras∙GTPγS molecule appears stably bound to Sos. The weak transient interaction between Sos and the second H-Ras∙GTPγS may provide a necessary mechanism for complex dissociation upon the completion of the native GDP → GTP exchange reaction, but also explains measurable GTP → GTP exchange activity of Sos routinely observed in *in vitro* assays that use fluorescently-labelled analogs of GTP. Overall, the data presents the first dynamic snapshot of Ras functional cycle as controlled by Sos.

Ras proteins are small GTPases that control several cellular signaling transduction pathways including proliferation, apoptosis and growth of cells^1^,^2^. Mutations to Ras have been implicated in around 30% of human tumors^3^. Ras acts as a molecular switch, cycling between active GTP- and inactive GDP- loaded forms. The activation (GTP-loading) of Ras is required for interaction with effector enzymes such as Raf. Ras has several functional regions that are involved in its interactions with downstream effector proteins. The P-loop (residues G10–S17) is responsible for phosphate binding, while the Switch I (Q25–Y40) and Switch II (D57–G75) regions are critical for interactions with guanine nucleotide exchange factors (GEF)^4^ and effector proteins^5^. The release of bound GDP from Ras is stimulated by the binding of a GEF Son of Sevenless (Sos), allowing GTP to bind in its place^6^,^7^. The Sos itself is activated, via the positive feedback mechanism, by Ras·GTP binding at its allosteric binding site, with this initial binding causing structural rearrangements within Sos^8^,^9^ that consequently lead to enhanced nucleotide exchange in the second Ras molecule bound at the catalytic site of Sos. The 57 kDa fragment of Sos (residues 563-1049, named Sos^Cat^), which is responsible for catalyzing this nucleotide exchange, consists of two essential domains, Ras exchange motif (REM) and Cdc25 domain - the latter comprising the catalytic site where Ras nucleotide exchange occurs. The allosteric site of Sos is located between the REM and Cdc25 domains, on the opposite (distal) side of the molecule to the catalytic site^8^,^9^. The structural snapshots provided by crystal structures of Ras:Sos complexes^8^,^10^ explain how the ternary functional complex is formed between the nucleotide-free Ras (Ras^NF^), Ras·GTP and Sos, however it remains to be established what triggers complex dissociation once the nucleotide exchange on Ras is complete, regenerating Sos for further binding reactions.

In its free form, Ras itself has been well known to experience a wide range of motions, depending on its nucleotide binding state, as revealed by solution NMR spectroscopy. Spectra of the GTP-loaded Ras have been affected by the intrinsic chemical exchange processes at intermediate rates on the NMR chemical shift timescale, with a number of signals broadened beyond detection^11^,^12^,^13^,^14^. Signal broadening and disappearance is predominantly observed for a few residues in the functionally important regions of the GTP-loaded H-Ras, such as P-loop, Switch I and Switch II regions. ^15^N-backbone relaxation measurements are able to report on internal motions of residues in these functionally important regions of H-Ras. Previous analysis of ^15^N-relaxation measurements showed dynamic residues that are involved in conformational exchange processes occurring throughout the G-domain (residues 1-166) of GTP-loaded H- and K-Ras^15^,^16^. Moreover, ^31^P NMR experiments have revealed that GTP-loaded H-Ras can undergo a dynamic equilibrium between two conformations, State 1 and State 2^17^,^18^. In addition, structural studies established that the bound nucleotide in the State 1 conformation has greater exposure to the surrounding solvent, allowing faster association and dissociation of GDP/GTP compared to State 2 and implies that State 1 is involved in guanine nucleotide exchange^19^,^20^. H-Ras binding to effector proteins, such as Raf, shifts the equilibrium towards the State 2 conformation. The solution structure and the dynamics of State 1 GTP-loaded H-Ras was determined by using H-RasT35S mutant in complex with stable GTP analog GppNHp^21^. Switch I and II loops in State 1 are dynamically mobile on the picosecond to nanosecond timescale, whereas these motilities are not apparent in the State 2 conformation^21^. In addition, the crystal structure of the nucleotide-free H-Ras (H-Ras^NF^) complex with Sos^Cat^ showed the opening of the nucleotide binding pocket of H-Ras, caused by a deviation of the Switch I loop^4^,^8^. The dynamic behavior of Ras in complex with Sos using NMR spectroscopy has not been described to date.

The native biological function of Sos is to catalyze heteronucleotide GDP → GTP exchange on Ras, however, many nucleotide exchange assays reported in the literature^8^,^10^,^14^,^22^,^23^,^24^,^25^,^26^ assess homonucleotide exchange rates (GTP → GTP^*^, where * marks differently labelled version of the same nucleotide) as a measure of Ras nucleotide exchange activity catalyzed by Sos. The previous studies, involving solution and crystallography characterization, revealed the presence of a stable ternary Ras·GTP:Sos:Ras^NF^ complex formed between Sos, GTP-loaded Ras (bound at its allosteric site), and nucleotide-free Ras (bound at the catalytic site)^8^,^10^. Recently it was shown that binding of Ras to a particular site on Sos depends heavily on the type of nucleotide loaded on Ras, making the process of nucleotide exchange much more selective and more directed than previously thought^7^. Ras·GTP preferentially binds to the allosteric site thus activating Sos, with only weak affinity for the catalytic site of activated Sos^7^. In contrast, Ras·GDP does not bind strongly to non-activated Sos and is unable to activate Sos itself, but binds strongly to the catalytic site of Sos once activated by Ras·GTP^7^. Therefore, it was suggested that Sos is much more active in promoting physiologically-relevant heteronucleotide GDP → GTP exchange when both Ras·GDP and Ras·GTP are present in solution. Despite these advances, the structural or dynamic properties of the hypothetical ternary Ras·GTP:Sos:Ras·GTP complex, which is expected to be present at the very end of native GDP → GTP exchange reaction, as well as during GTP → GTP* reaction commonly used in *in vitro* assays, has yet to be described in significant detail. Here, we explore whether H-Ras, loaded with a stable non-hydrolizable GTP analog (GTPγS, guanosine 5′-O-[gamma-thio]triphosphate), can form a stable stoichiometric 2:1 H-Ras:Sos^Cat^ complex in solution. We also describe the effective dynamic changes on H-Ras (derived from measured heteronuclear relaxation parameters R_1,_ R_2_ and ^15^N{^1^H}NOEs for amide groups) associated with overall binding to Sos^Cat^. Although these changes cannot be ascribed specifically to allosteric or catalytic site binding, these measurements reveal significant differences in the internal motions of the P-loop and Switch regions between free H-Ras·GTPγS and H-Ras·GTPγS mixed with Sos^Cat^ at a 2:1 stoichiometry, with the binding process triggering a significant change in protein dynamics, making many regions much more flexible in the complex, which may weaken nucleotide binding and explain low-level activity of Sos for homonucleotide exchange. Overall, both from the NMR dynamics measurements and SEC-MALS analysis, the complex appears to be transient: Ras·GTPγS effectively occupies only one of the two potential binding sites on Sos^Cat^, while binding only transiently at the second binding site. These data further demonstrate that Ras·GTP should dissociate from Sos once the native nucleotide exchange is complete, and supports our previous suggestion that passive homonucleotide GTP → GTP* exchange observed in *in vitro* assays relies on transient protein interactions.

## Materials and Methods

### Protein expression and purification

As described previously^7^, DNA constructs coding for H-Ras (residues 1-166) and the functional fragment of Sos (Sos^Cat^, residues 563-1049) were synthesized by Geneart (Life Technologies) and sub-cloned into a pET28b vector, which has been modified to include the TEV cleavage site after the N-terminal His-tag sequence. Proteins were expressed in BL21-GOLD (DE3) *E. coli* competent cells and the seeder cultures were grown in Luria Broth (LB) medium. Uniformly [^15^N,^2^H]-labelled Sos^Cat^ and H-Ras were obtained by growing bacteria in 2 L of M9 minimal media in D_2_O containing 1 g/L [^15^N]-ammonium chloride, 2 g/L of D_7_-Glucose, 50 μg/mL of kanamycin and 12.5 μg/mL of tetracycline antibiotics, supplemented with micronutrients and vitamins. Cells were incubated at 250 rpm at 37 °C and then cooled to 25 °C at an OD_600_ of 0.6–0.8. Cells were then induced by 0.1 mM of IPTG followed by the additional 2 g/L feed of D_7_ Glucose in each flask. Each flask was incubated at 25 °C, 250 rpm for a further 24 hours. The unlabeled proteins were produced similarly using non-labelled media components. All proteins were cleaved from their purification tags via TEV protease^7^. Protein purification and GTPγS loading on H-Ras was carried out as described previously^7^. Purified H-Ras samples were incubated with 20 fold excess of GTPγS and 1/100 fold of His-tagged Sos in 50 mM Hepes, 50 mM NaCl, 2 mM MgCl_2_, 2 mM TCEP, 0.1 mM EDTA, 0.02% NaN_3_ at pH 7.4. The samples were left overnight at 4 °C to allow nucleotide exchange. The mixture was then passed through a Ni-NTA column equilibrated with 30 mM Na_2_HPO_4_, 1 mM DTT, 2 mM MgCl_2_ and 0.1 mM EDTA, 0.02% NaN_3_ at pH 7.0 to remove the His_6_-tagged Sos and free nucleotide. The samples were concentrated using 0.5 mL VivaSpin concentrators (Sartorius, 10,000 MWCO). Protein concentration was determined by standard Bradford assays. Nucleotide exchange was confirmed by electrospray mass spectrometry.

### Backbone dynamics

All NMR relaxation experiments were carried out on the Bruker 800 MHz Avance III spectrometer equipped with TCI cryoprobe. A total of four samples were used for measurements, free [^15^N,^2^H]-labelled 400 μM H-Ras·GTPγS or 200 μM Sos^Cat^ (“free samples”), or 200 μM H-Ras·GTPγS mixed with 100 μM Sos^Cat^, where one of the proteins was [^15^N,^2^H]-labelled and the other unlabelled (at 2:1 stoichiometry, which we will call here “mixed samples”). All NMR experiments were recorded in 30 mM sodium phosphate buffer pH 7.5, 1 mM DTT, 2 mM MgCl_2_, 0.1 mM, EDTA, 0.02% NaN_3_, with 5% D_2_O added for lock. Changes in relaxation parameters were recorded for samples using labelled H-Ras·GTPγS and Sos^Cat^ at 290 K and 298 K, respectively. TROSY-based R_1_, R_1ρ_ and ^15^N{^1^H}NOE experiments were carried out using the pulse sequences as described in published protocols^27^. For each of the R_1_ values, the order of the relaxation delays was randomized. R_1_ experiments were recorded with time delays of 0, 200, 400, 640, 1000, 1280, 1600 and 2000 ms. R_1ρ_ experiments were recorded with time delays of 8, 32, 64, 96, 128 and 160 ms. R_2_ rates were derived from our R_1ρ_ measurement using the formulae as described by Lakomek *et al*.^27^. The ^15^N{^1^H}NOE experiments were carried out by recording a pair of spectra with and without proton saturation. NOE values were then analyzed by calculating the peak height ratios obtained from reference and ^1^H-saturated experiments (I_sat_/I_ref_). To independently determine the correlation time τ_c_, TROSY-based (^15^N) ŋ_xy_ experiments^28^ were recorded for free and mixed samples with time delays of 1, 32, 64, 96, 128 and 160 ms. The derived τ_c_ (which we denote here τ_c_^ŋxy^) was calculated using home-built Matlab scripts, based on a well-established approach^28^,^29^. All relaxation data were processed and analyzed with NMRPipe^30^. Model-free analysis of heteronuclear relaxation parameters and measurement of τ_c_ derived from R_2_/R_1_ ratio (τ_c_^R2/R1^) was conducted using TENSOR2 software^31^. The expected values of τ_c_ for the rigid-body approximations (τ_c_^rigid^) were calculated using HYDRONMR software^32^ based on known static 3D structures of proteins or of their complexes.

### SEC and SEC-MALS

The stoichiometry of the H-Ras·GTPγS:Sos interaction in mixed samples was first assessed using Superdex 75 Size Exclusion Chromatography (SEC) column. Sec-MALS experiments were obtained for a sample of 200 μM Sos^Cat^ mixed with 400 μM H-Ras·GTPγS with a flow rate of 0.75 mL/min, an injection volume of 200 μL and using a 50 mM Hepes buffer pH 7.5, 50 mM NaCl, 2 mM MgSO_4_, 2 mM TCEP, 0.1 mM EDTA, 0.02% NaN_3_ at 25 °C. The protein content of each SEC fraction (500 μL) was checked by loading 20 μL samples on polyacrylamide gel, and staining with Coomassie blue. H-Ras:Sos mixed samples were also characterized by SEC coupled with Multi-Angle Light Scattering (SEC-MALS). A stoichiometric 2:1 mixture of H-Ras·GTPγS with Sos^Cat^ was injected onto a 24 mL Superdex X200 column. Sample eluting from the column passed through an in-line Jasco UV-2077 Plus UV detector (280 nm absorbance), a DAWN HELEOS-II laser photometer (laser wavelength 658 nm) and an Optilab rEX refractometer with a QELS dynamic light scattering attachment (all from Wyatt Technology). Light scattering intensity and eluent refractive index (concentration) were analyzed using ASTRA v6.0.6 software to yield a weight-averaged molecular mass (Mw).

## Results

### Spectral changes in H-Ras and Sos^Cat^ associated with complex formation

To capture the core of the Ras-Sos interaction yet minimizing the size of the system, the functionally-competent protein fragments were used. H-Ras residues 1-166 (which we will refer to as simply H-Ras) encompasses the essential fully-functional G-domain, only lacking the C-terminal tail that links Ras to the membrane. To avoid unwanted hydrolysis of unstable GTP nucleotide during experiments, we used the slowly hydrolysable analogue, GTPγS^14^,^33^, which was pre-loaded on H-Ras at 1:1 ratio. This analogue has been previously shown to match more closely the GTP-binding properties of small GTPases than the frequently used analogue GMPPNP/GppNHp^14^,^34^,^35^,^36^. GTPγS was also used in our earlier study to disentangle site-specific and nucleotide-specific preferences of H-Ras binding to Sos^7^. No spectral changes were observed during the course of the experiments reported here, or previously^7^ which could be attributed to any significant GTPγS hydrolysis for the chosen experimental conditions. The fragment of Sos used here, Sos^Cat^, encompasses the two essential modules, Ras exchange motif (REM) domain, and the Cdc25 domain; it contains both allosteric and catalytic binding sites for Ras and is fully sufficient to promote nucleotide exchange in Ras^8^. A 2:1 H-Ras·GTPγS:Sos^Cat^ mixture is expected to represent and model the equilibrium state of a particular functional stoichiometric complex in the Ras-Sos functional cycle where Sos is fully-activated by the allosteric site binding and the native nucleotide exchange on Ras molecule bound at the catalytic site has just been completed, with the H-Ras present at the catalytic site loaded with GTP (mimicked by GTPγS in our study). This should enable us to determine if both H-Ras·GTPγS molecules remain bound to Sos^Cat^ in this situation, or only one of them, or neither. From the viewpoint of H-Ras, binding can occur in principle to two different binding sites, allosteric and catalytic. Thus, the NMR spectra of H-Ras is inherently expected to contain a mixture of these two binding states, complicating a binding-site-specific interpretation. Therefore, the questions that we pose here for this model can be formulated as: (1) how the effective overall dynamics of H-Ras·GTPγS is affected by the presence of Sos^Cat^; (2) how stable is this hypothetical ternary complex; and (3) what is the effective binding stoichiometry in this case.

**Figure 1:**
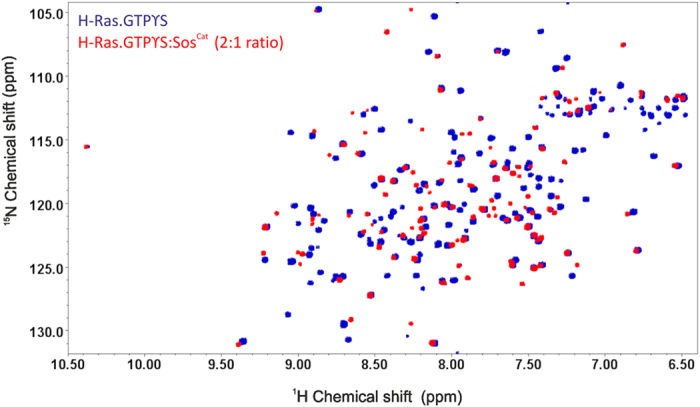
Overlay of TROSY spectra of [^15^N,^2^H]-H-Ras·GTPγS in free form (blue) and mixed with unlabeled Sos^Cat^ at 2:1 ratio (red).

**Figure 2:**
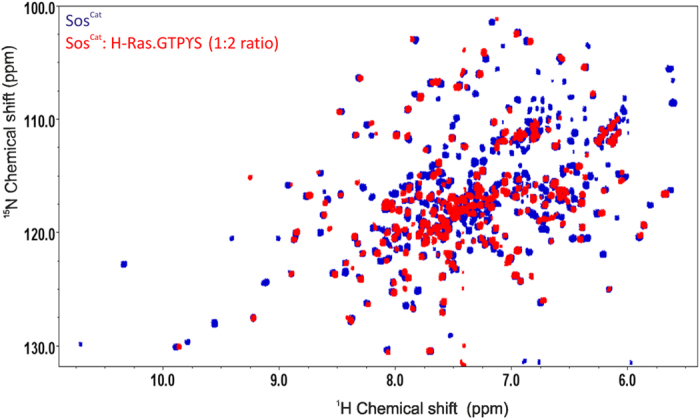
Overlay of TROSY spectra of [^15^N,^2^H]-labelled Sos^Cat^ in free form (blue) and mixed with unlabeled H-Ras·GTPγS at 1:2 ratio (red).

To explore the changes in H-Ras spectra and dynamics associated with the complex formation, first we have recorded ^1^H-^15^N-correlation TROSY NMR spectra for the free [^15^N,^2^H]-labelled H-Ras·GTPγS, as well as for the 2:1 mixture of labelled H-Ras·GTPγS with non-labelled Sos^Cat^. Superimposing these spectra revealed significant changes in amide signal positions of H-Ras, evident of the complex formation (Fig. 1). Similar superposition was also done previously for spectra acquired at slightly higher temperature, which lead to similar results^7^. Although a number of Ras signals remained unperturbed, many signals shifted and/or were broadened beyond detection in the mixed sample (see Supplemental Figure S1 for residue-specific analysis of signal shifts). Notably for the mixture, H-Ras is in the fast exchange regime^7^, with no evidence for a pattern of signals from unbound H-Ras present, suggesting that all H-Ras present in the sample is involved in binding, at least transiently. Furthermore, we have recorded ^1^H-^15^N-TROSY spectra of [^15^N,^2^H]-labelled Sos^Cat^ in the free form, and with addition of two equivalents of non-labelled H-Ras·GTPγS (Fig. 2). The spectral comparison again reveals extensive spectral perturbations, this time from the viewpoint of Sos, signifying complex formation. Interestingly, the majority of visible Sos peaks appear not to be greatly influenced by the expected increase in protein complex size, with only some resonances, presumably originating from residues at the binding interface, affected by transient binding between Sos^Cat^ and H-Ras·GTPγS. The lack of sequence-specific assignments of Sos^Cat^, challenging due to the large size of this protein even in its unbound form, unfortunately prevented any residue-specific analysis from the viewpoint of Sos^Cat^. Partial assignments of H-Ras·GTPγS cross-peaks in ^1^H-^15^N-TROSY spectra in free and bound state was however achieved by matching and transferring the known assignments of H-Ras·GMPPNP at pH 7.5^11^,^14^,^37^ for the peaks which retained similar positions and patterns in the ^1^H-^15^N-correlation spectra (see Supplemental Table S1). Overall, approximately 70% of the peak assignments could be transferred with reasonable degree of confidence to the ^1^H,^15^N- TROSY spectra of the free H-Ras·GTPγS and in complex with Sos^Cat^ (see Supplemental Figure S2). The overall good spectral quality, and the number of peaks retaining their positions in complex, were somewhat surprising for the expected 96 kDa size of the ternary complex (see below), however even incomplete transfer of signal assignments allowed reasonable sampling of dynamic changes along the length of the polypeptide chain of H-Ras upon addition of Sos^Cat^. This enabled us to record a suite of NMR experiments for characterizing the dynamics of H-Ras at a residue-specific level, as well as for measuring the overall H-Ras and Sos^Cat^ tumbling rates for free and mixed samples.

### Comparison of backbone dynamics of H-Ras·GTPγS in free form and when mixed with Sos^Cat^

**Figure 3:**
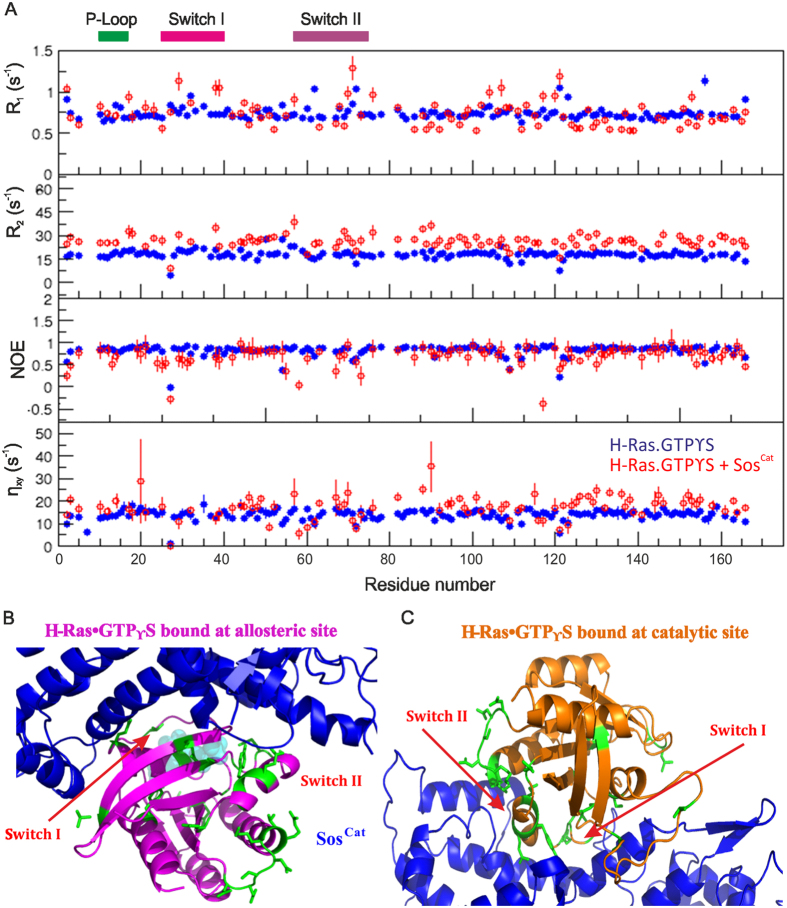
Changes in relaxation parameters for amide groups of H-Ras·GTPγS upon binding with Sos^Cat^. (**A**) Plots of R_1_, R_2_, ^15^N{^1^H} NOE and η_xy_ for the residues in the free H-Ras·GTPγS (blue) and in complex with Sos^Cat^ (red). Only data for assigned residues are included. Positions of P-Loop, Switch I and Switch II regions are shown on the top and labelled accordingly. Residues displaying significant change in relaxation parameters upon binding are mapped onto the crystal structure (PD ID: 1NVW) of H-Ras:Sos complex, indicated in green, with positions of allosteric (**B**) and catalytic (**C**) sites expanded.

To assess the change in mobility of the functionally-important regions of [^15^N,^2^H]-labelled H-Ras·GTPγS upon complex formation with Sos^Cat^, the backbone dynamics were first studied for free protein by measuring R_1_, R_2_ rates and ^15^N{^1^H} NOEs for amide groups, using standard well-established approach^27^,^29^,^38^,^39^,^40^,^41^. Plotting the values of the measured relaxation parameters (Fig. 3A) showed that residues Q25-T35 from the Switch I and E62-T74 from the Switch II regions, as well as K104-E107, A121-R123 and F156 exhibit lower values of R_2_ and ^15^N{^1^H}NOE in contrast to higher R_1_ values, that can be interpreted as large-amplitude motions (increased flexibility) on the sub-nanosecond time scale. A high rigidity throughout most of the remaining residues is clearly evident from rather uniform R_1_, R_2_ and ^15^N{^1^H}-NOE values. The backbone dynamics observed for residues K104-E107, A121-R123 and F156 in the H-Ras·GTPγS were almost identical to those of H-Ras·GDP and H-RasT35S·GppNHp mutant previously studied by others^21^.

**Figure 4:**
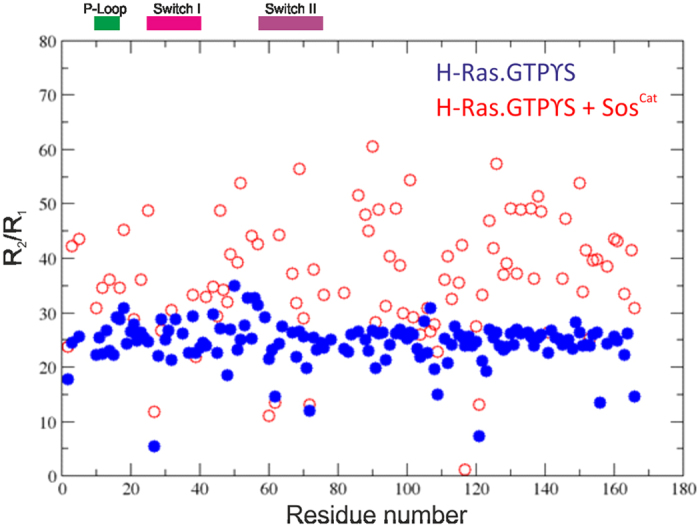
R_2_/R_1_ ratio for free H-Ras·GTPγS (blue) and bound with Sos^Cat^ (red). Positions of P-Loop, Switch I and Switch II regions are shown on the top and labelled accordingly.

Measuring heteronuclear relaxation parameters, as above, for the [^15^N,^2^H]-labelled H-Ras·GTPγS mixed with Sos^Cat^ enabled direct comparison of free *vs* effective “bound” state on a per-residue basis (Fig. 3A). It should be noted here that this effective bound state includes the mixture of two distinct binding states, allosteric and catalytic sites of Sos^Cat^, with possible binding in fast exchange on the NMR timescale, which makes it difficult to ascribe these changes to binding at a particular binding site^7^. Nevertheless, the relaxation behavior of H-Ras in this mixed sample suggested noticeably different internal motions from those observed for free H-Ras. It was apparent that no obvious population of free H-Ras remained in the mixture. In addition, contrary to the intuitively expected formation of a large and static complex, for example as seen in the crystal structure of H-Ras·GppNp:Sos^Cat^:H-Ras^NF^ (pdb ID: 1NVW^8^) involving nucleotide-free H-Ras bound at the catalytic site, our data showed only a small global increase in the R_2_ values, less than 2-fold (Fig. 3A), which may indicate only a marginal increase in the apparent molecular size of the NMR-visible population of H-Ras in the mixed sample, suggesting that the complex is highly dynamic. Compared to the free H-Ras·GTPγS, there is an overall redistribution of apparent dynamics throughout the H-Ras backbone when complexed with Sos, as is evident from the R_2_/R_1_ ratios (Fig. 4). Approximately 90% of R_2_/R_1_ values range between 20–28 for free H-Ras·GTPγS, which increases to 25–47 for the complex. This observed wider distribution of R_2_/R_1_ values indicates that not all amide groups become more rigid, and that numerous sites within H-Ras in complex with Sos retain their mobility, with some even increasing mobility.

Using ^15^N{^1^H}NOE as a probe for fast internal dynamics in the picosecond to nanosecond timescales, it is clear that most functionally relevant regions of H-Ras·GTPγS showed markedly enhanced mobility (average ^15^N{^1^H}NOE dropping from ~0.8 to ~0.6, Fig. 3A) upon complex formation with Sos^Cat^. In particular, low ^15^N{^1^H}NOE values have been detected in the P-loop (involved in nucleotide binding), Switch-II regions and in the helices between the two Switch regions, which are expected to be in close vicinity to the Sos allosteric and catalytic sites (Fig. 3B). These effects are also visible as an increase in the R_1_ values especially for residues S17, I21 (in and around the P-loop), H27, T38, S39 (in the Switch I region), G48, D57, Q70, Y71, M72, E76 and E91 (in and around the Switch II region), compared to the un-complexed H-Ras·GTPγS. Further regions (residues R102-D108, K117, L120, A121 and E153) also exhibit slightly slower internal motions compared to that of free H-Ras·GTPγS (Fig. 3A) indicating small changes to Ras dynamics throughout the core of the protein upon binding Sos.

Independently, amide ^15^N,^1^H-cross-correlated relaxation rates (η_xy_)^28^ were measured for the free H-Ras·GTPγS and when mixed with Sos^Cat^ (bottom panel in Fig. 3A). Comparison of cross-correlated rates for free and complexed states reveal re-arrangements in local mobility, but without a significant overall increase in η_xy_, as would be expected for a large and static complex. Taken together, all the heteronuclear relaxation data point to a fairly dynamic complex formed between H-Ras·GTPγS and Sos^Cat^ when these components are present in a 2:1 stoichiometry. The data also shows increased flexibility and dynamics occurring in the regions of H-Ras involved in nucleotide binding; it is difficult however to discriminate from this data whether these dynamic changes originate from the binding of H-Ras to the catalytic or allosteric sites, or both.

### Measuring the rotational correlation time for H-Ras·GTPγS and Sos proteins

The size of molecular assembly has a clear effect on its molecular tumbling rate. To investigate the molecular tumbling of the H-Ras·GTPγS protein in free and bound state, the TROSY-based ŋ_xy_ experiment^28^,^29^ was used to measure the overall rotational correlation time (denoted τ_c_^ŋxy^). The effective τ_c_^ŋxy^ measured for free H-Ras·GTPγS is ~11 ns, as expected for a 19 kDa globular protein (Table 1) which suggests that the nucleotide bound H-Ras is monomeric and well folded in solution. Surprisingly, the H-Ras·GTPγS in complex with Sos^Cat^ showed only a marginal increase in the effective rotational correlation time. The measured value for τ_c_^ŋxy^ of ~17 ns is substantially lower than what is expected from the hydrodynamics calculations for the static binary or ternary complex with Sos^Cat^ (see Table 1).

**Figure 5:**
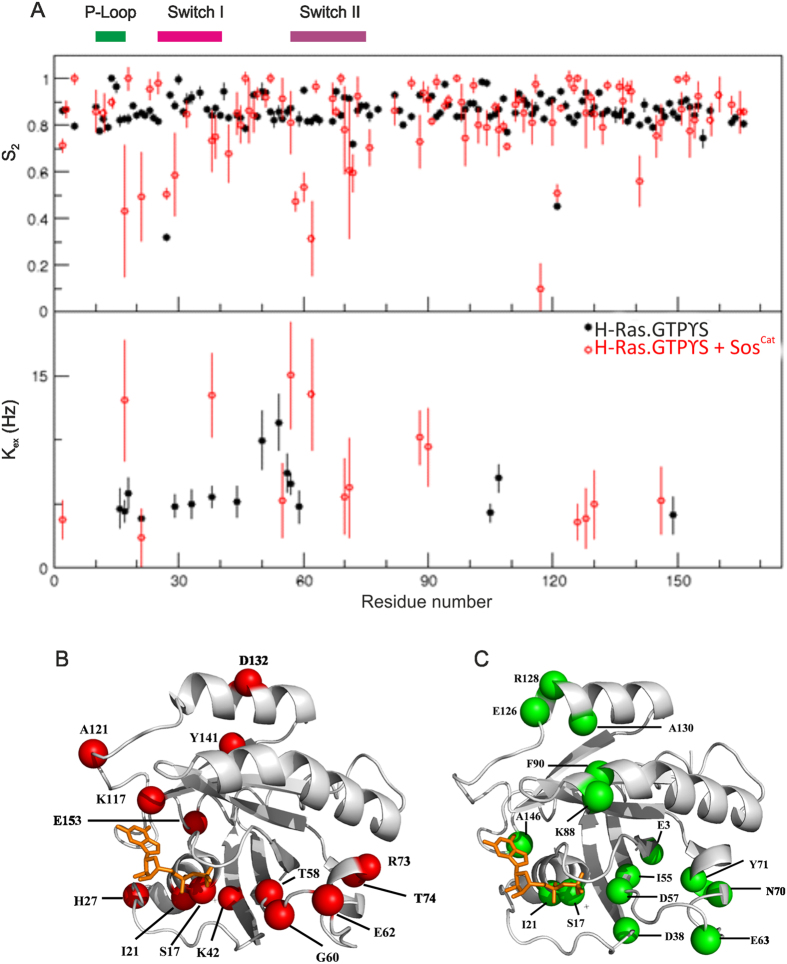
Plots of calculated S^2^ values and chemical exchange rate (k_ex_) values verses amino acid sequence of H-Ras. (**A**) The S^2^ and k_ex_ values (calculated using Tensor V.2 software) of free H-Ras·GTPγS (black) and complexed with Sos^Cat^ (red) were plotted against residue number. Positions of P-Loop, Switch I and Switch II regions are shown on the top and labelled accordingly. Residues demonstrating significant differences in S^2^ (**B**) and k_ex_ (**C**) upon Sos^Cat^ binding are shown as red and green spheres, respectively, on the crystal structure of H-Ras.

The somewhat surprising discrepancy between the experimentally-observed rotational correlation time τ_c_^ŋxy^ of H-Ras·GTPγS in complex with Sos, and that expected for a static stoichiometric complex, can be reasonably explained by the general fluidity of the complex, and the presence of a H-Ras·GTPγS population that is only loosely bound to Sos^Cat^. Overall, in the fast exchange regime, the effective τ_c_ of an ensemble of the higher-order complexes should be given by the population-weighted average of the τ_c_ of the different conformations. This is also in line with the observation that the general quality of the 2D TROSY spectra for H-Ras·GTPγS:Sos^Cat^ 2:1 mixed samples, both from the viewpoint of H-Ras (Fig. 1) and Sos (Fig. 2) is much better than what can be usually expected for a static 96 kDa complex. To verify this observation further, the τ_c_^ŋxy^ was measured for [^15^N,^2^H]-Sos^Cat^ samples mixed with H-Ras·GTPγS. While the observed effective rotational correlation time τ_c_^ŋxy^ for unbound Sos is well within the range expected for a molecule of 57 kDa, the value measured in the mixed sample (~38 ns) was found to be only slightly larger than that of free Sos (~30 ns) (Table 1). The observed τ_c_^ŋxy^ value (38 ns) falls significantly short of the 68 ns expected for the static ternary H-Ras:Sos complex, but is close to τ_c_^rigid^ of 40 ns expected for 1:1 complex (estimates based on HYDRONMR calculations^32^, Table 1). As a control, we have also measured τ_c_^ŋxy^ for free Sos^Cat^W729E mutant, and when mixed with H-Ras·GTPγS (Table 1). This mutant is known to prevent H-Ras binding at the allosteric site of Sos^10^, and indeed, both of these τ_c_^ŋxy^ values were not significantly different, and close to the expected value for a free protein, suggesting that this mutant does not form a stable complex with H-Ras·GTPγS. The τ_c_^ŋxy^ value (38 ns) measured for the H-Ras·GTPγS:Sos complex was fairly close to values τ_c_^R2/R1^ of 36 ns obtained using an alternative approach, the model-free analysis using TENSOR2 software^31^, which derives the overall τ_c_ based on the experimental ^15^NH -relaxation rates (R_2_ and R_1_). It is to be noted that τ_c_^R2/R1^ obtained on [^15^N,^2^H]-H-Ras·GTPγS:Sos 2:1 sample was ~36 ns, whereas the observed τ_c_^ŋxy^ for this sample was only ~17 ns (Table 1). This discrepancy may come from the fact that the ^15^N CSA-dipole dipole cross relaxation rates obtained in ŋ_xy_ experiment are independent of any chemical exchange contributions, and therefore provide the lower limit estimate of the overall tumbling correlation time τ_c_^29^. The chemical exchange contributions, which mainly affect the R_2_ rates, however are not completely excluded when τ_c_^R2/R1^ is calculated from R_2_/R_1_, leading to an overestimated value, compared to τ_c_^ŋxy^. The large number of residues affected by significant k_ex_ contributions from chemical or conformation exchange for the mixed sample is evident from Fig. 5C (see below), and is consistent with transient complex formation. Overall, from these comparisons (Table 1), it is likely that the lower than expected experimental values of τ_c_ for the mixed samples observed here using two different independent methods can be explained by the formation of a dynamic transient complex between H-Ras·GTPγS and Sos^Cat^.

### Model-free analysis of H-Ras·GTPγS dynamics

A further quantitative residue-specific evaluation of ^15^N relaxation data was performed using the model-free approach, employing the program TENSOR2^31^, for unbound H-Ras·GTPγS and H-Ras·GTPγS mixed with Sos^Cat^. Most H-Ras residues in the mixed sample show large variations in their S^2^ values (S^2^ describes the motional rigidity of the system), which suggests that the system is highly fuzzy and could not be easily described by the fitted model. This can be illustrated by a significant number of high χ^2^ values obtained in the process of automated model selection by TENSOR2 which were scattered throughout the whole polypeptide chain, in comparison to the free H-Ras·GTPγS where larger χ^2^ values were mainly localized in the flexible loop regions (see Supplemental Figure S3).

The P-loop, Switch I and Switch II regions of the H-Ras·GTPγS:Sos complex display a high degree of internal mobility, with most residues in the P-loop region exhibiting an average S^2^ value of ~0.5. Residues of the Switch II region in the H-Ras:Sos complex exhibit similar internal motions to the P-loop with an average S^2^ value of ~0.6, see Fig. 5A,B. Residues in the Switch I region display slightly less flexibility compared to the P-loop and Switch II region with an average S^2^ value of ~0.7. In contrast, the free H-Ras·GTPγS appears to be more rigid (S^2^ = ~0.8) than H-Ras·GTPγS mixed with Sos (Fig. 5), suggesting that the internal dynamics for most of the regions in H-Ras·GTPγS occur on the picosecond to nanosecond timescales.

As an alternative measure of the apparent residue-specific change in dynamics, chemical exchange contributions to the transverse relaxation rates were evaluated by rate constants (k_ex_) for free and complexed H-Ras·GTPγS (Fig. 5A). For the majority of the observable residues in the free H-Ras·GTPγS the k_ex_ values were nearly zero suggesting that these residues have very little contributions from conformational exchange. However, residues near the Switch II region (residues E49, D54, L56-A59) have moderate contributions to the chemical exchange rate in the range of up to ~10 Hz indicating that these residues are undergoing chemical exchange in the millisecond time scales (Fig. 4A). For the H-Ras·GTPγS mixed with Sos, a significant chemical or conformational exchange contribution to relaxation was observed for residues E3, S17, I21, D38, I55, D57, E62, Q70, Y71, K88, F90, E126, R128, A130 and A146 (Fig. 5C), suggesting wide-spread dynamic motions throughout the protein. Overall the average k_ex_ values are slightly higher than those of free H-Ras·GTPγS, suggesting that the complex undergoes chemical exchange or a conformational process upon interactions with Sos, which again may be indicative of a transient binding process. Taken together, the model-free approach has revealed that the functionally important regions of H-Ras are inherently flexible. More importantly, the results show greater flexibility in some H-Ras regions upon binding to Sos, with extensive conformational rearrangements on a micro to millisecond timescale, that demonstrates that the complex formed has a dynamic nature. Increased chemical exchange contributions to the transverse relaxation rates k_ex_ suggest a presence of slower dynamic processes, all consistent with transient complex formation. Although it has not been possible to interpret the local changes in the H-Ras polypeptide chain dynamics in the context of binding to a specific site on Sos, allosteric or catalytic, the increased motions within the nucleotide-binding regions of H-Ras (P-loop, Switch I and Switch II) upon addition of Sos^Cat^ may provide a mechanism for loosening nucleotide binding and thus possibly explains enhanced homonucleotide GTP → GTP exchange observed in the presence of Sos^8^,^10^,^14^,^22^,^23^,^24^,^25^,^26^.

### Analysis of the apparent H-Ras·GTPγS:Sos^Cat^ complex stoichiometry using SEC

**Figure 6:**
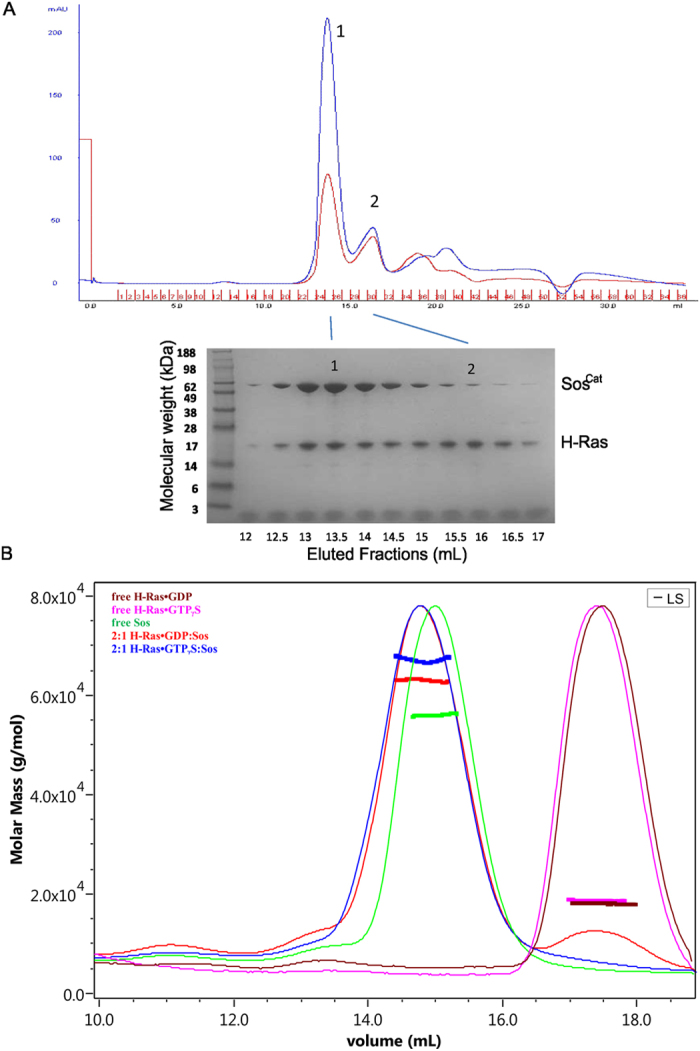
Analysis of the H-Ras:Sos complex by SEC and SEC-MALS. (**A**) Size exclusion chromatography (SEC) profile of 2:1 H-Ras·GTPγS:Sos^Cat^ mixture loaded on the column. UV detection traces at 280 nm (blue) and 254 nm (red) are shown. Peaks 1 and 2 correspond to H-Ras:Sos complex, and mostly unbound H-Ras, respectively. The protein component content of each individual fraction, as marked, was analyzed by PAGE. (**B**) Overlay of SEC-MALS traces, as detected by the eluant’s differential refractive index and scaled by the maximum peak intensity, of free H-Ras preloaded with GTPγS or GDP, free Sos^Cat^, and 2:1 mixtures of Ras:Sos loaded on the column, as labelled. Molecular weight (in g/mol, or Da) of each point of the peak was determined by MALS.

The transient H-Ras·GTPγS interaction with Sos^Cat^ suggested above differs from the apparent stability of Ras·GppNp:Sos:Ras^NF^ complex, previously successfully isolated by Size Exclusion Chromatography (SEC) and used for crystallography studies^8^. Both of these complexes involve activated Sos^Cat^, but the major difference between these complexes is nucleotide-loading state of H-Ras molecule present at the catalytic site of Sos, a difference which may have significant effect on the complex stability. To check whether the complex formed by GTPγS-loaded H-Ras with Sos^Cat^ is stable enough to withstand purification on a SEC column, a 2:1 H-Ras·GTPγS:Sos mixture was loaded, and eluted fractions collected and assessed. The data shows (Fig. 6) that despite its somewhat fluid nature, the H-Ras·GTPγS:Sos complex formed can be successfully isolated and detected (as peak 1) in SEC fractions (Fig. 6A). The peak 2 eluted around 16 mL corresponded largely to the unbound H-Ras, which is likely to have dissociated from Sos in the course of the chromatographic run. The protein content of each elution fraction was further analyzed by PAGE, and the presence of both Sos and H-Ras is evident as upper (57 kDa) and lower bands (19 kDa) on the gel (Fig. 6A). Interestingly, although all Sos apparently co-eluted with H-Ras, significant proportion of H-Ras eluted without Sos. The peak 2, corresponding to mostly unbound H-Ras, likely to have dissociated from the complex, nevertheless contained very small amounts of Sos (around 5% of total protein in the fraction, Fig. 6A). This result shows that although proteins were loaded on the column as stoichiometric 2:1 H-Ras·GTPγS:Sos mixture, around half of all H-Ras (judging by the band intensities on the gel, Fig. 6A) has dissociated from Sos and eluted separately.

To measure further the molecular sizes of the protein species eluted when 2:1 H-Ras·GTPγS:Sos mixture was loaded on a column, we employed SEC coupled with static multi-angle light scattering (MALS), a technique also used previously to characterize H-Ras·GppNp:SosCat:H-RasNF ternary complex^8^,^42^. Control SEC-MALS experiments were performed on H-Ras·GDP and H-Ras·GTPγS in the absence of Sos^Cat^ and, as expected, the molecular weights were 18.2 ± 0.1 and 18.8 ± 0.1 kDa, respectively (Fig. 6B). Not surprisingly, MALS measurements for Sos alone showed the molecular weight to be 56.9 ± 0.3 kDa, as expected for this 57 kDa protein. In the control experiment, the apparent molecular weight measured for the eluted H-Ras·GDP:Sos^Cat^ complex was only 63.6 ± 0.6 kDa when these proteins were loaded on the column as a 2:1 mixture. This value is lower than 76 kDa expected for 1:1 complex, however it matches the previous observation that H-Ras·GDP binds Sos^Cat^ only weakly, with *K_d_* of around 54 μM, causing only very weak spectral perturbations observed in the NMR spectra for this complex^7^. For a similar H-Ras·GTPγS:Sos^Cat^ mixture the apparent size of the eluted complex was 68.5 ± 1.5 kDa which was reasonably close but slightly lower than 76 kDa expected for 1:1 complex (Fig. 6B). This matches with the previously measured tighter binding of these proteins (overall macroscopic *K_d_* of around 5 μM), and observation that Ras·GTPγS binds mostly at the allosteric site of Sos^Cat^ 7. These results suggest that the complexes between H-Ras·GTPγS and Sos, as well as between H-Ras·GDP and Sos, are not stable enough to retain 2:1 stoichiometry during passage through the chromatographic column. This observation is in line with NMR results on τ_c_ presented above (Table 1), suggesting that the effective stoichiometry of H-Ras·GTPγS:Sos^Cat^ complex appears to be close to 1:1. The ternary complex between H-Ras·GTPγS and Sos therefore is highly fluid and dynamic in nature, and relies on transient interactions. This complex is expected to be formed in the course of normal functional cycle immediately after the native GDP → GTP exchange reaction is completed, and the inherent weakness of such a complex suggests a mechanism for Ras·GTP release and Sos recycling for the subsequent catalytic reactions.

## Discussion

Previous X-Ray crystallography studies^8^ have established that the ternary complex formed between H-Ras loaded with a GTP analog (GppNp), Sos^Cat^ and nucleotide-free H-Ras (H-Ras^NF^) is stable and persists as 2:1 stoichiometric assembly that can be purified as such on a size-exclusion chromatographic column^8^,^42^. The total mass of the complex as detected by SEC-MALS is similar to the mass expected for this ternary complex^8^,^42^. Such protein assembly presumably represents a stalled transition complex in the catalytic cycle of Sos, when bound nucleotide-free Ras “waits” for a GTP molecule to come along and bind to complete the nucleotide exchange reaction. Here, we have studied in detail the immediate next stage of the functional cycle, when H-Ras bound at the catalytic site finally acquires GTP, and there are two Ras·GTP molecules present per one Sos molecule. This state was modelled here, in the equilibrium, by mixing two equivalents of H-Ras·GTPγS with one equivalent of Sos^Cat^. The questions which we have addressed are: what is the dynamic state of this complex, does it retain rigidity, or does this complex dissociate? One intuitively expected outcome of this state is that the complex would be destabilized at this point and will be dissociating, to recycle Sos for further catalytic reactions (which is a striking difference in behavior compared to the ternary complex containing one nucleotide-free Ras). However, this is also a state giving an appreciable exchange rate for GTP → GTP reactions as measured in numerous nucleotide exchange assays^8^,^10^,^14^,^22^,^23^,^24^,^25^,^26^, implying at least some Ras·GTP binding at the catalytic site of Sos, to effect this exchange reaction. Moreover, recently it was proposed that the rate of such exchange is further modulated by the dynamic fluctuations in the complex^25^, with such fluctuations inconsistent with a static complex. In our recent study we also explored the site-specific and nucleotide-specific binding affinities of Ras and Sos^Cat^ and demonstrated that although the overall apparent *K*_*d*_ is around 5 μM for Ras·GTPγS binding to Sos^Cat^ according to fluorescence-detected titrations, Ras·GTPγS binds strongly only to the allosteric site (with expected *K*_*d*_ ≤ 5 μM), but very weakly to the catalytic site of activated Sos^Cat^, with *K*_*d*_ of around 21 μM, suggesting that Ras·GTP should be released from the catalytic site once the native nucleotide exchange is complete^7^. Our data presented here explores this further, and show that this hypothetical ternary Ras·GTPγS:Sos:Ras·GTPγS complex is highly dynamic, and is likely to exist as a fast-interconverting pool of conformers. Although it was not possible to discern from our data unequivocally whether the changes in dynamics are caused by allosteric or catalytic site binding, it is reasonable to assume that the observable H-Ras signals were dominated by a species in the fast exchange with the catalytic site^7^. From the previous^7^ and present studies it emerges that the first H-Ras·GTP molecule preferentially binds to the allosteric site of Sos, with the second molecule binding to Sos at the catalytic site only transiently. The presence of such dynamic transient complex can reconcile the apparent contradiction between, on one hand, a functional requirement for Sos to dissociate from Ras·GTP and be recycled for the following GDP → GTP catalytic exchange reactions, and, on another hand, the appreciative homonucleotide GTP → GTP exchange widely observed in the assays. As we demonstrated here, the mere presence of Sos^Cat^ makes H-Ras·GTP more dynamically fluid, which can promote nucleotide release and passive homonucleotide exchange *in vitro*. However *in vivo*, the native heteronucleotide GDP → GTP exchange would rely on specific H-Ras·GDP recognition by the activated Sos, and hence lead to much higher exchange efficiency^7^.

We present here three lines of evidence to show that the complex formed by Sos^Cat^ with Ras·GTP is dynamic and transient: (i) there is no overall significant signal broadening in the NMR spectra of the protein mixtures, neither from the viewpoint of Ras nor Sos, with fast chemical exchange on the NMR timescale observed for the complex; (ii) the values of correlation times τ_c_ from both Ras and Sos when in complex, derived from the cross-correlation relaxation rates and R_2_/R_1_ ratios, are consistent with each other and are characteristic for a 1:1 complex, but are clearly not compatible with 2:1 rigid complex; and (iii) despite being able to co-elute Ras·GTPγS and Sos^Cat^ from the SEC column, the static light scattering data on the eluted peaks shows the averaged molecular weight of the species present in the co-eluted peak matches a 1:1 complex. The apparent less-than-1:1 stoichiometry of H-Ras·GDP:Sos^Cat^ complex revealed here by SEC-MALS in the control experiment also fits with the observation that H-Ras·GDP interacts only weakly with the catalytic binding site of Sos, and not with the allosteric site^7^. Transient binding of H-Ras·GTP and Sos^Cat^ may provide the mechanism for switching Sos to the distinctive active states observed recently in single-molecule studies^25^. The transient complex between Ras·GTPγS and Sos^Cat^ observed here may be best described as a fuzzy protein complex^43^.

The detailed comparison of heteronuclear relaxation parameters measured for assigned amide signals of H-Ras·GTPγS in unbound form, and when mixed with Sos^Cat^, revealed how the changes in dynamics associated with this overall transient complex formation are distributed along the H-Ras sequence, despite not being able to correlate these unambiguously with the allosteric or catalytic site binding on Sos^Cat^. Previous dynamic studies of H-Ras and K-Ras loaded with stable mimics of GTP revealed conformational dynamics in the millisecond timescale that involved residues in the P-loop, Switch II and other regions^15^,^16^. In addition, R_1_ and NOE measurements of the GppNHp-bound H-Ras involving rapid internal motion (on the picosecond-to-nanosecond timescale) suggested that the conformational changes upon effector binding and nucleotide exchange are limited to residues 31-42 (Switch I), 61-75 (Switch II), 107-109 and 121-123. Our R_1_ and NOE measurements of H-Ras·GTPγS show similar dynamic motions to those obtained for H-Ras·GppNHp^21^ demonstrating that these GTP mimics have similar effects on H-Ras. However, our R_1_ measurements also reveal further dynamic changes to residues 14, 15 (P-loop), 27, 30 (Switch I) and 156 that were less obvious^21^ or were missing in previous analysis of GTP-loaded H-Ras^11^. These slight differences in dynamics are probably due to the varying construct lengths, the choice of GTP mimic^36^ and different pH conditions in our and previous studies. The X-ray structure of H-Ras·GppNHp (PDB ID: 4EFL) reveals these residues are situated near the nucleotide-binding site and are therefore likely to be affected by the change in nucleotide. Furthermore, faster dynamics of GDP-loaded H-Ras has been examined on the sub-nanosecond timescale and revealed differences between the G-domain of H-Ras (residues 1-166) and full length H-Ras (1-189) in dynamics of the P-loop, Switch I and II regions^44^,^45^. The data obtained in these studies for H-Ras·GTPγS corroborates well with the other published data^11^,^21^,^44^,^45^ and with our relaxation measurements, which show for the first time that the functionally important regions of H-Ras responsible for the nucleotide binding undergo the most significant dynamic changes in response to interactions with Sos. This change in local protein dynamics upon binding may lead to loosening of nucleotide binding and hence explain the observed “catalytic” effect of Sos on promoting passive nucleotide exchange (e.g., GTP → GTP*) driven by an inherent excess of one nucleotide species (e.g., GTP*) present in solution. Previously we suggested that in addition to this relatively inefficient “passive” mechanism which relies on weak transient interactions, more efficient “active” mechanism will work for the native GDP → GTP exchange which would rely on the tight and targeted binding of Ras·GDP to the catalytic site of the activated Sos, with subsequent fast release of Ras·GTP from the catalytic site once the exchange is complete, followed by another tight Ras·GDP binding, etc.^7^.

In conclusion, the present study provides further evidence that the stability and stoichiometry of the complex between Ras and Sos strongly depends on the type of nucleotide which is loaded on Ras. Unlike a relatively stable stoichiometric ternary complex formed by H-Ras·GTP, Sos^Cat^ and H-Ras^NF^ 8,42, the ternary H-Ras·GTP:Sos:H-Ras·GTP complex is very dynamic and transient, at least at one of the two binding sites. The transient nature of this complex provides a mechanism for fast dissociation following the successful physiological GDP → GTP nucleotide exchange on Ras bound at the catalytic site, thus regenerating Sos for the next stage of its functional cycle. At the same time, the efficiency of the homonucleotide GTP → GTP exchange catalyzed by this complex (e.g., when fluorescently-labelled GTP is exchanged for non-labelled GTP, or vice versa) may not truly represent the native catalytic function of Sos, due to only transient binding of Ras to Sos at the catalytic site^7^. Further experiments will be useful in the future to explore the dynamics specifically in relation to allosteric and catalytic site binding, for example using mutants blocking particular binding sides, and using differential labelling. The work reported here presents a first dynamic snapshot of Ras functional cycle, and reveals for the first time that Ras:Sos complex can be dynamic, as well as static, at various stages of its functional cycle, depending on the type of nucleotide bound. These results contribute towards fundamental understanding the activation of Ras via its interactions with Sos. Better overall understanding of this system may speed up development of new anti-cancer treatments.

## Additional Information

**How to cite this article**: Vo, U. *et al*. Dynamic studies of H-Ras·GTPγS interactions with nucleotide exchange factor Sos reveal a transient ternary complex formation in solution. *Sci. Rep.* **6**, 29706; doi: 10.1038/srep29706 (2016).

## Supplementary Material

Supplementary Information

## Figures and Tables

**Table 1:** Rotational correlation times determined for H-Ras and Sos^Cat^ and their selected complexes.

Protein(s) in the sample	τ_c_^ŋxy^, ns from ŋ_xy_	τ_c_^R2/R1^, ns from TENSOR2	τ_c_^rigid^, ns from HYDRONMR calculations
[^15^N,^2^H]-H-Ras·GTPγS	11 ± 0.7	11	11.8 (3TGP)
[^15^N,^2^H]-H-Ras·GTPγS + Sos^Cat^, 2:1 ratio	17 ± 2	36.4	68 (1NVW)
[^15^N,^2^H]-Sos^Cat^	30 ± 10	N/A	31 (2II0)
[^15^N,^2^H]-Sos^Cat^W729E	32 ± 3	N/A	N/A
[^15^N,^2^H]-H-Ras·GTPγS + Sos^Cat^, 1:1 ratio	N/A	N/A	40 (1KBD)
[^15^N,^2^H]-Sos^Cat^ + H-Ras·GTPγS, 1:2 ratio	38.4 ± 8	N/A	68 (1NVW)
[^15^N,^2^H]-Sos^Cat^W729E + H-Ras·GTPγS, 1:2 ratio	32 ± 5	N/A	N/A

The table shows comparison between τ_c_ measured experimentally using ŋ_xy_ cross-relaxation experiments^29^ (τ_c_^ŋxy^), based on calculating R_2_/R_1_ ratio using TENSOR2 program^46^ (τ_c_^R2/R1^), and those calculated for “rigid structure” (τ_c_^rigid^) using HYDRONMR software^32^ based on known static 3D structures of proteins or their complexes (with PDB IDs used shown in brackets). N/A labels cells where the information was not obtained or not available. Both TENSOR2 and HYDRONMR base their τ_c_ calculation on the assumption of a static molecular structure.

## References

[b1] Buday L. & Downward J. Many faces of Ras activation. Biochim Biophys Acta 1786, 178–187, 10.1016/j.bbcan.2008.05.001 (2008). 18541156

[b2] Cherfils J. & Zeghouf M. Regulation of small GTPases by GEFs, GAPs, and GDIs. Physiol Rev 93, 269–309, 10.1152/physrev.00003.2012 (2013). 23303910

[b3] Pylayeva-Gupta Y., Grabocka E. & Bar-Sagi D. RAS oncogenes: weaving a tumorigenic web. Nature Rev Cancer 11, 761–774, 10.1038/nrc3106 (2011). 21993244 PMC3632399

[b4] Boriack-Sjodin P. A., Margarit S. M., Bar-Sagi D. & Kuriyan J. The structural basis of the activation of Ras by Sos. Nature 394, 337–343, 10.1038/28548 (1998). 9690470

[b5] Downward J. Targeting RAS signalling pathways in cancer therapy. Nature Rev Cancer 3, 11–22, 10.1038/nrc969 (2003). 12509763

[b6] Rojas J. M., Oliva J. L. & Santos E. Mammalian son of sevenless Guanine nucleotide exchange factors: old concepts and new perspectives. Genes Cancer 2, 298–305, 10.1177/1947601911408078 (2011). 21779500 PMC3128634

[b7] Vo U. . Monitoring Ras Interactions with the Nucleotide Exchange Factor Son of Sevenless (Sos) Using Site-specific NMR Reporter Signals and Intrinsic Fluorescence. J Biol Chem. 291, 1703–1718, 10.1074/jbc.M115.691238 (2016). 26565026 PMC4722452

[b8] Margarit S. M. . Structural evidence for feedback activation by Ras.GTP of the Ras-specific nucleotide exchange factor SOS. Cell 112, 685–695, 10.1016/s0092-8674(03)00149-1 (2003). 12628188

[b9] Freedman T. S. . A Ras-induced conformational switch in the Ras activator Son of sevenless. Proc Natl Acad Sci USA 103, 16692–16697, 10.1073/pnas.0608127103 (2006). 17075039 PMC1629002

[b10] Sondermann H. . Structural analysis of autoinhibition in the Ras activator Son of sevenless. Cell 119, 393–405, 10.1016/j.cell.2004.10.005 (2004). 15507210

[b11] Ito Y. . Regional polysterism in the GTP-bound form of the human c-Ha-Ras protein. Biochemistry 36, 9109–9119, 10.1021/bi970296u (1997). 9230043

[b12] Hu J. S. & Redfield A. G. Conformational and dynamic differences between N-ras P21 bound to GTPgammaS and to GMPPNP as studied by NMR. Biochemistry 36, 5045–5052, 10.1021/bi963010e (1997). 9125526

[b13] Fenwick R. B. . Solution Structure and Dynamics of the Small GTPase RalB in its Active Conformation: Significance for Effector Protein Binding. Biochemistry 48, 2192–2206, 10.1021/bi802129d (2009). 19166349

[b14] Smith M. J., Neel B. G. & Ikura M. NMR-based functional profiling of RASopathies and oncogenic RAS mutations. Proc Natl Acad Sci USA 110, 4574–4579, 10.1073/pnas.1218173110 (2013). 23487764 PMC3607025

[b15] O’Connor C. & Kovrigin E. L. Global conformational dynamics in ras. Biochemistry 47, 10244–10246, 10.1021/bi801076c (2008). 18771285

[b16] Buhrman G. . Analysis of binding site hot spots on the surface of Ras GTPase. J Mol Biol 413, 773–789, 10.1016/j.jmb.2011.09.011 (2011). 21945529 PMC3247908

[b17] Spoerner M., Herrmann C., Vetter I. R., Kalbitzer H. R. & Wittinghofer A. Dynamic properties of the Ras switch I region and its importance for binding to effectors. Proc Natl Acad Sci USA 98, 4944–4949, 10.1073/pnas.081441398 (2001). 11320243 PMC33143

[b18] Spoerner M., Nuehs A., Herrmann C., Steiner G. & Kalbitzer H. R. Slow conformational dynamics of the guanine nucleotide-binding protein Ras complexed with the GTP analogue GTPgammaS. FEBS J. 274, 1419–1433, 10.1111/j.1742-4658.2007.05681.x (2007). 17302736

[b19] Shima F. . Structural basis for conformational dynamics of GTP-bound Ras protein. J Biol Chem 285, 22696–22705, 10.1074/jbc.M110.125161 (2010). 20479006 PMC2903345

[b20] Liao X. . Hypermethylation of RAS effector related genes and DNA methyltransferase 1 expression in endometrial carcinogenesis. International Journal of Cancer 123, 296–302, 10.1002/ijc.23494 (2008). 18404674

[b21] Araki M. . Solution structure of the state 1 conformer of GTP-bound H-Ras protein and distinct dynamic properties between the state 1 and state 2 conformers. J Biol Chem. 286, 39644–39653, 10.1074/jbc.M111.227074 (2011). 21930707 PMC3234787

[b22] Ford B., Skowronek K., Boykevisch S., Bar-Sagi D. & Nassar N. Structure of the G60A mutant of Ras: implications for the dominant negative effect. J Biol Chem 280, 25697–25705, 10.1074/jbc.M502240200 (2005). 15878843

[b23] Gureasko J. . Role of the histone domain in the autoinhibition and activation of the Ras activator Son of Sevenless. Proc Natl Acad Sci USA 107, 3430–3435, 10.1073/pnas.0913915107 (2010). 20133692 PMC2816639

[b24] Kunzelmann S. & Webb M. R. Fluorescence detection of GDP in real time with the reagentless biosensor rhodamine-ParM. Biochemical J. 440, 43–49, 10.1042/BJ20110349 (2011). PMC3204873 21812760

[b25] Iversen L. . Molecular kinetics. Ras activation by SOS: allosteric regulation by altered fluctuation dynamics. Science 345, 50–54, 10.1126/science.1250373 (2014). 24994643 PMC4255705

[b26] Burns M. C. . Approach for targeting Ras with small molecules that activate SOS-mediated nucleotide exchange. Proc Natl Acad Sci USA 111, 3401–3406, 10.1073/pnas.1315798111 (2014). 24550516 PMC3948241

[b27] Lakomek N. A., Ying J. & Bax A. Measurement of 15N relaxation rates in perdeuterated proteins by TROSY-based methods. J Biomol NMR 53, 209–221, 10.1007/s10858-012-9626-5 (2012). 22689066 PMC3412688

[b28] Lee D., Hilty C., Wider G. & Wuthrich K. Effective rotational correlation times of proteins from NMR relaxation interference. J Magn Reson 178, 72–76, 10.1016/j.jmr.2005.08.014 (2006). 16188473

[b29] Lakomek N. A. . Internal dynamics of the homotrimeric HIV-1 viral coat protein gp41 on multiple time scales. Ang Chemie 52, 3911–3915, 10.1002/anie.201207266 (2013). PMC3610801 23450638

[b30] Delaglio F. . NMRPipe: a multidimensional spectral processing system based on UNIX pipes. J Biomol NMR 6, 277–293, 10.1007/bf00197809 (1995). 8520220

[b31] Dosset P., Hus J. C., Blackledge M. & Marion D. Efficient analysis of macromolecular rotational diffusion from heteronuclear relaxation data. J Biomol NMR 16, 23–28, 10.1023/a:1008305808620 (2000). 10718609

[b32] Garcia de la Torre J., Huertas M. L. & Carrasco B. HYDRONMR: prediction of NMR relaxation of globular proteins from atomic-level structures and hydrodynamic calculations. J Magn Reson 147, 138–146, 10.1006/jmre.2000.2170 (2000). 11042057

[b33] Karim A. M. & Thompson R. C. Guanosine 5′-O-(3-Thiotriphosphate) as an Analog of Gtp in Protein-Biosynthesis - the Effects of Temperature and Polycations on the Accuracy of Initial Recognition of Aminoacyl-Transfer Rna Ternary Complexes by Ribosomes. J Biol Chem 261, 3238–3243 (1986). 3512549

[b34] Gasmi-Seabrook G. M. . Real-time NMR study of guanine nucleotide exchange and activation of RhoA by PDZ-RhoGEF. J Biol Chem 285, 5137–5145, 10.1074/jbc.M109.064691 (2010). 20018869 PMC2820740

[b35] Mazhab-Jafari M. T. . Real-time NMR study of three small GTPases reveals that fluorescent 2′(3′)-O-(N-methylanthraniloyl)-tagged nucleotides alter hydrolysis and exchange kinetics. J Biol Chem 285, 5132–5136, 10.1074/jbc.C109.064766 (2010). 20018863 PMC2820739

[b36] Long D. . A comparative CEST NMR study of slow conformational dynamics of small GTPases complexed with GTP and GTP analogues. Ang Chemie 52, 10771–10774, 10.1002/anie.201305434 (2013). 24039022

[b37] Gossert A. D., Hiller S. & Fernandez C. Automated NMR resonance assignment of large proteins for protein-ligand interaction studies. J Am Chem Soc 133, 210–213, 10.1021/ja108383x (2011). 21162534

[b38] Kay L. E. Protein dynamics from NMR. Nat Struct Biol 5 Suppl, 513–517, 10.1038/755 (1998). 9665181

[b39] Zidek L., Novotny M. V. & Stone M. J. Increased protein backbone conformational entropy upon hydrophobic ligand binding. Nat Struct Biol 6, 1118–1121 (1999). 10581552 10.1038/70057

[b40] Sahu S. C., Bhuyan A. K., Udgaonkar J. B. & Hosur R. V. Backbone dynamics of free barnase and its complex with barstar determined by N-15 NMR relaxation study. J Biomol NMR 18, 107–118, 10.1023/A:1008310402933 (2000). 11101215

[b41] Stone M. J. NMR relaxation studies of the role of conformational entropy in protein stability and ligand binding. Acc Chem Res 34, 379–388, 10.1021/ar000079c (2001). 11352716

[b42] Sondermann H., Zhao C. & Bar-Sagi D. Analysis of Ras:RasGEF interactions by phage display and static multi-angle light scattering. Methods 37, 197–202, 10.1016/j.ymeth.2005.05.016 (2005). 16288886

[b43] Tompa P. & Fuxreiter M. Fuzzy complexes: polymorphism and structural disorder in protein-protein interactions. Trends Biochem Sci 33, 2–8, 10.1016/j.tibs.2007.10.003 (2008). 18054235

[b44] Thapar R., Williams J. G. & Campbell S. L. NMR characterization of full-length farnesylated and non-farnesylated H-Ras and its implications for Raf activation. J Mol Biol 343, 1391–1408, 10.1016/j.jmb.2004.08.106 (2004). 15491620

[b45] Kraulis P. J., Domaille P. J., Campbell-Burk S. L., Van Aken T. & Laue E. D. Solution structure and dynamics of ras p21.GDP determined by heteronuclear three- and four-dimensional NMR spectroscopy. Biochemistry 33, 3515–3531 (1994). 8142349 10.1021/bi00178a008

[b46] Mandel A. M., Akke M. & Palmer A. G. 3rd. Dynamics of ribonuclease H: temperature dependence of motions on multiple time scales. Biochemistry 35, 16009–16023, 10.1021/bi962089k (1996). 8973171

