# Supplementary material for: Dynamic studies of H-Ras•GTPγS interactions with nucleotide exchange factor Sos reveal a transient ternary complex formation in solution
Source: Sci Rep. 2016 Jul 14;6:29706. doi: 10.1038/srep29706 (PMC4944212; doi:10.1038/srep29706)
Supplement: Supplementary Information [file srep29706-s1.pdf]

Supplemental data

Dynamic studies of H-Ras•GTPγS interactions with nucleotide exchange factor Sos reveal a transient ternary complex formation in solution

Uybach Vo[1], Navratna Vajpai[2], Kevin J. Embrey [2,*], and Alexander P. Golovanov [1,*]

[1] Manchester Institute of Biotechnology and Faculty of Life Sciences, The University of Manchester, 131 Princess Street, Manchester M1 7DN, UK, [2] AstraZeneca, Discovery Sciences, Mereside, Alderley Park, Cheshire, SK10 4TF, UK

[*] Correspondence should be addressed to Alexander P. Golovanov (email: A.Golovanov@manchester.ac.uk) and Kevin J. Embrey (email: Kevin.Embrey@astrazeneca.com)

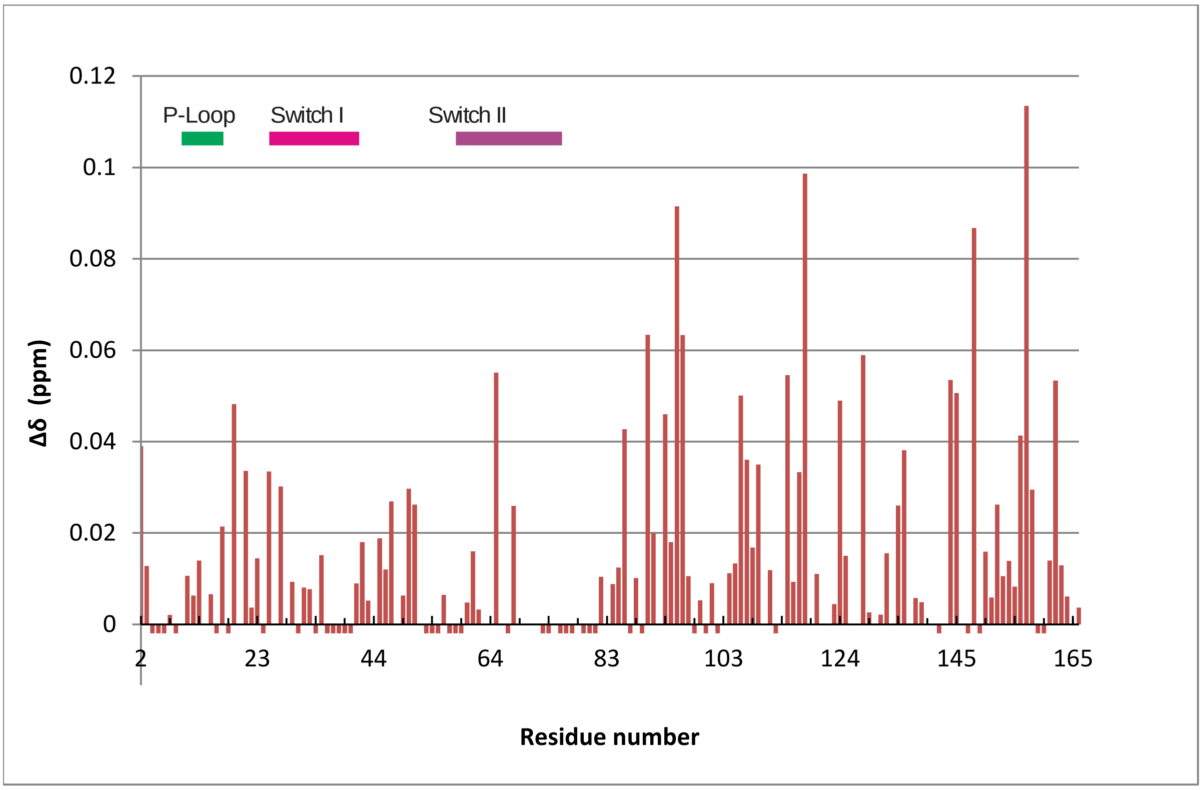

Figure S1. NMR chemical shift perturbations $\Delta\delta$ upon addition of Sos^{Cat} to [^{15}N,^{2}H]-H-Ras•GTPγS (2:1 Ras:Sos ratio) for amide signals of Ras assigned to specific amino acid residues. The small negative bars mark sequence position of residues where signals were not assigned. Functional regions of Ras are annotated. The perturbations were calculated as $\Delta\delta = \sqrt{(\delta_f^H - \delta_m^H)^2 + (\frac{\delta_f^N - \delta_m^N}{10})^2}$, where superscripts H and N denote chemical shifts in ^{1}H and ^{15}N dimensions, respectively, and subscripts f and m denote free and mixed state.

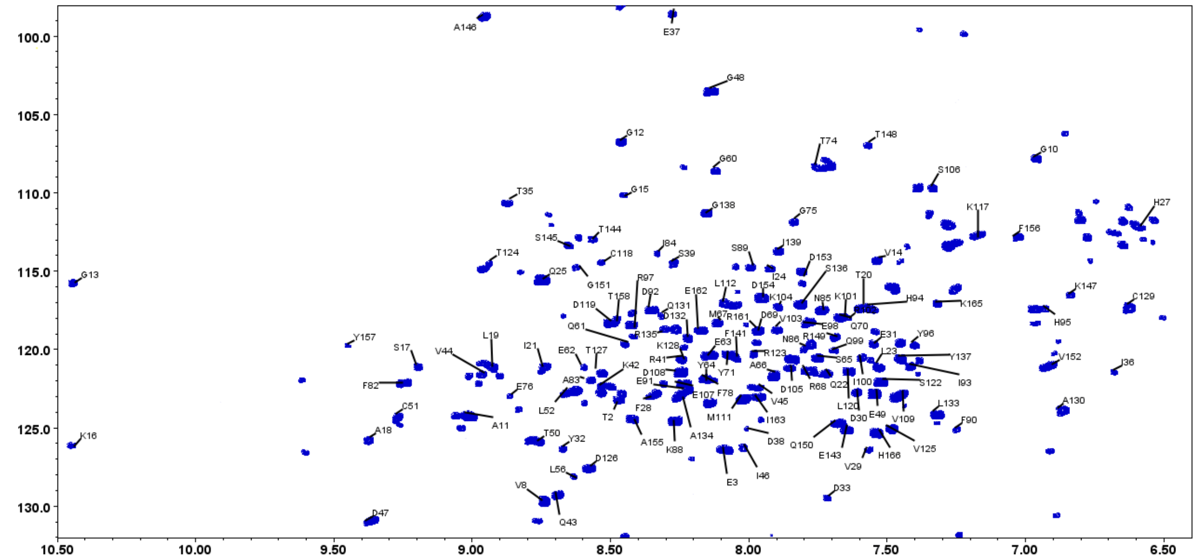

Figure S2. ^{1}H-^{15}N-TROSY spectrum of [^{15}N,^{2}H]-H-Ras•GTPγS with transferred amide signal assignments, as labelled.

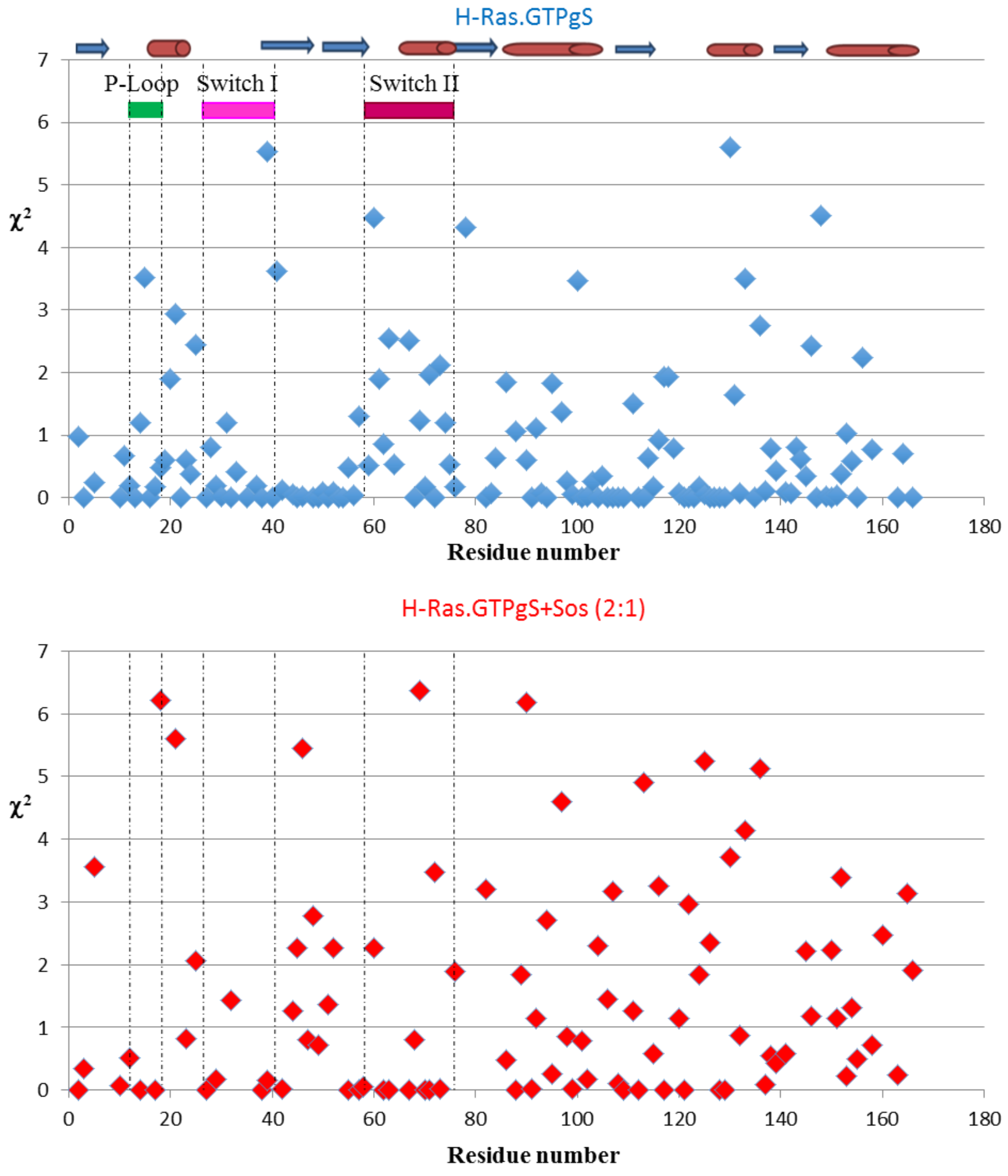

Figure S3. The values of χ^2 calculated by TENSOR2 following automatic model selection for unbound H-Ras•GTPγS (blue, top graph) and H-Ras•GTPγS mixed with Sos^{Cat} (red, bottom graph), versus residue number. The secondary structure of Ras is indicated by arrows (β-sheets) and cylinders (α-helices). Functional regions of Ras are annotated. H-Ras•GTPγS:Sos complex is affected by transient interactions, leading to generally poor fit throughout the whole H-Ras•GTPγS sequence.

Table S1. Partial assignments of H-Ras•GTPγS cross-peaks in ^{1}H-^{15}N-TROSY spectra in free and Sos-bound state achieved by matching and transferring the known assignments of H-Ras•GMPPNP at pH 7.5 (Ito et al, 1997; Smith et al, 2013; Gossert et al, 2011) for the peaks which retained similar positions and patterns in the ^{1}H-^{15}N-correlation spectra.

Residue number	Amino acid name	H-RasGTPgS		H-RasGTPgS:Sos 2:1	
		δ^{H}	δ^{N}	δ^{H}	δ^{N}
2	T	8.48	123.41	8.47	123.03
3	E	8.09	126.55	8.10	126.50
8	V	8.81	129.36	8.80	129.36
10	G	6.96	107.88	6.96	107.88
11	A	9.02	124.32	9.01	124.37
12	G	8.46	106.80	8.47	106.75
13	G	10.44	115.67	10.44	115.81
14	V	7.54	114.35	7.54	114.35
15	G	8.44	110.13	8.44	110.06
17	S	9.20	121.09	9.19	120.88
19	L	8.92	121.15	8.97	120.97
20	T	7.56	117.43	7.56	117.43
21	I	8.74	121.22	8.73	120.92
22	Q	7.72	121.58	7.72	121.61
23	L	7.54	121.13	7.54	121.27
25	Q	8.86	116.57	8.83	116.71
26	N	7.89	117.32	7.89	117.32
27	H	6.61	112.01	6.58	112.18
28	F	8.35	122.87	8.35	122.87
29	V	7.58	126.44	7.57	126.35
31	E	7.55	119.72	7.56	119.78
32	Y	8.68	126.37	8.67	126.41
35	T	8.87	110.62	8.87	110.77
41	R	8.24	120.78	8.24	120.70
42	K	8.52	122.46	8.51	122.34
43	Q	8.69	129.35	8.70	129.32
44	V	8.96	121.61	8.96	121.61
45	V	7.91	121.54	7.91	121.73
46	I	8.02	126.22	8.02	126.33
47	D	9.39	131.09	9.38	130.88
48	G	8.14	103.55	8.14	103.55
49	E	7.54	122.77	7.55	122.72
50	T	8.76	126.00	8.79	125.83
51	C	9.27	124.29	9.27	124.55
52	L	8.64	122.73	8.64	122.73
56	L	8.63	128.11	8.63	128.05
60	G	8.12	108.57	8.13	108.59
61	Q	8.45	119.71	8.46	119.56
62	E	8.60	121.17	8.60	121.14
63	E	8.06	120.47	8.06	120.47
64	Y	8.16	121.88	8.16	121.88

65	S	7.75	120.67	7.70	120.88
66	A	8.05	123.45	8.05	123.45
68	R	7.79	121.37	7.77	121.47
69	D	8.04	120.62	8.04	120.62
70	Q	7.59	117.45	7.59	117.45
71	Y	8.08	120.39	8.08	120.39
74	T	7.77	108.38	7.77	108.38
78	F	8.03	123.13	8.03	123.13
82	F	9.26	122.20	9.25	122.13
83	A	8.63	122.88	8.63	122.88
84	I	8.33	113.73	8.34	113.81
85	N	7.73	117.62	7.73	117.50
86	N	7.81	119.99	7.78	119.68
88	K	8.28	124.58	8.27	124.63
90	F	7.25	125.23	7.29	125.77
91	E	8.25	122.24	8.23	122.18
92	D	8.36	117.47	8.36	117.47
93	I	7.42	121.07	7.39	120.69
94	H	7.48	116.20	7.48	116.03
95	H	7.32	117.08	7.26	116.40
96	Y	7.40	119.80	7.46	119.51
97	R	8.43	118.51	8.43	118.40
99	Q	7.69	120.20	7.70	120.24
101	K	7.68	118.06	7.67	117.97
103	V	7.90	118.72	7.90	118.72
104	K	7.96	118.84	7.97	118.82
105	D	7.85	121.18	7.86	121.28
106	S	7.34	109.76	7.39	109.74
107	E	8.22	122.62	8.24	122.90
108	D	8.26	121.57	8.25	121.46
109	V	7.45	122.89	7.48	123.09
111	M	7.99	123.21	7.99	123.21
112	V	7.85	120.60	7.84	120.65
114	V	9.04	128.53	9.04	128.53
115	G	7.99	114.78	8.05	114.77
116	N	8.57	122.05	8.57	121.96
117	K	7.19	112.76	7.16	112.69
118	C	8.63	114.84	8.53	114.51
119	D	8.50	118.39	8.50	118.39
120	L	7.64	121.48	7.63	121.43
121	A	8.02	123.19	8.02	123.19
122	A	7.53	122.08	7.53	122.08
123	R	7.76	120.51	7.76	120.54
124	T	8.94	114.50	8.96	114.91
125	V	7.49	125.17	7.48	125.04
126	E	8.57	127.63	8.57	127.63
127	S	8.54	121.53	8.54	121.53
128	R	8.47	118.01	8.42	117.68

129	C	6.63	117.41	6.63	117.42
130	A	6.87	123.85	6.87	123.85
131	Q	8.32	117.89	8.32	117.89
132	D	8.31	122.09	8.32	122.20
133	L	7.32	124.15	7.32	124.15
134	A	7.96	122.43	7.99	122.44
135	R	8.31	118.79	8.27	118.76
136	S	7.79	118.36	7.79	118.36
137	Y	7.45	120.60	7.46	120.65
138	G	8.15	111.41	8.15	111.37
139	I	7.90	113.76	7.90	113.76
141	Y	8.15	120.55	8.15	120.55
143	E	7.64	125.14	7.64	125.14
144	T	8.62	112.98	8.57	112.85
145	S	8.70	113.18	8.65	113.29
146	A	8.97	132.67	8.97	132.67
148	T	7.57	107.06	7.49	106.69
150	Q	7.69	124.76	7.67	124.73
151	G	8.74	115.66	8.75	115.64
152	V	6.93	121.16	6.91	121.01
153	E	7.81	117.15	7.82	117.18
154	D	7.96	116.83	7.97	116.74
155	A	8.43	124.55	8.43	124.47
156	F	7.08	112.70	7.04	112.91
157	Y	9.44	119.68	9.33	119.42
158	T	8.09	117.05	8.07	117.15
161	R	8.22	119.36	8.23	119.22
162	E	7.97	118.63	8.02	118.57
163	I	7.97	123.14	7.97	123.02
164	Q	8.18	118.83	8.18	118.80
165	Q	7.90	117.95	7.90	117.95
166	H	7.54	125.41	7.54	125.39
